# An Immersive Virtual Kitchen Training System for People with Multiple Sclerosis: A Development and Validation Study

**DOI:** 10.3390/jcm12093222

**Published:** 2023-04-30

**Authors:** Massimiliano Pau, Eleonora Cocco, Federico Arippa, Giulia Casu, Micaela Porta, Shay Menascu, Anat Achiron, Alon Kalron

**Affiliations:** 1 Department of Mechanical, Chemical and Materials Engineering, University of Cagliari, 09124 Cagliary, Italy; massimiliano.pau@unica.it (M.P.); federico.arippa@unica.it (F.A.); giuliacasu.gc@outlook.it (G.C.); micaela.porta@unica.it (M.P.); 2 Multiple Sclerosis Centre, Department of Medical Sciences and Public Health, University of Cagliari, 09124 Cagliari, Italy; ecocco@unica.it; 3 Multipe Sclerosis Center, Sheba Medical Center, Tel-Hashomer 52621, Israel; shay.menascu@sheba.health.gov.il (S.M.); anat.achiron@sheba.health.gov.il (A.A.); 4 Sackler Faculty of Medicine, Tel-Aviv University, Tel-Aviv 69978, Israel; 5 Sagol School of Neurocience, Tel-Aviv University, Tel-Aviv 69978, Israel; 6 Department of Physical Therapy, School of Health Professions, Sackler Faculty of Medicine, Tel-Aviv University, Tel-Aviv 69978, Israel

**Keywords:** immersive, virtual reality, rehabilitation, feasibility, upper limb, multiple sclerosis

## Abstract

Rehabilitation via virtual reality (VR) training tools allows repetitive, intensive, and task-specific practice in a controlled and safe environment. Our goal was to develop and validate a novel immersive VR system based on the practice of real-life activities in a kitchen environment in people with multiple sclerosis (pwMS) with upper-limb dysfunction. The novel immersive VR kitchen application includes several tasks, i.e., tidying up the kitchen, preparing a hamburger and soup meal, and dish washing. Following the development phase, the system was tested for an 8-week intervention period on a small sample of pwMS suffering from upper-limb dysfunction. The Suitability Evaluation Questionnaire for VR systems served as the primary outcome. The scores for enjoyment, sense of comfort with the system, feelings of success and control, realism, easy-to-understand instructions, assists in rehabilitation therapy, were between 4.0 and 4.6, indicating a high satisfaction. The scores for eye discomfort, dizziness, nausea, and disorientation during practice were between 2.8 and 1.3, indicating a low-to-moderate interference of the system. The virtual kitchen training system is feasible and safe for upper-limb training in pwMS and paves the way for future RCTs to examine the benefits of the system compared with standard care, thus improving the functionality of the upper limbs in pwMS.

## 1. Introduction

Multiple sclerosis (MS), a chronic autoimmune disease of the central nervous system (CNS), is characterized by inflammation, demyelination, and neurodegeneration [1]. Damage to the CNS leads to a wide range of symptoms including muscle weakness, spasticity, fatigue, cognitive impairments, mobility difficulties, and vision problems, all negatively affecting quality of life [2]. Currently, there is no cure for MS; hence, various pharmacological and non-pharmacological treatments are essential in assisting with the management of the disease’s symptoms [3,4].

The percentage of people with multiple sclerosis (pwMS) with upper-limb dysfunction ranges from 50 to 76% [5,6,7]. Upper-limb dysfunction, one of the most common symptoms in essential daily life activities, consequently, reduces the quality of life. Notably, exercise and rehabilitation have been shown to be effective in improving upper-limb function in pwMS [8]. Nociti et al. and Spooren et al. confirmed that upper-limb training interventions (e.g., constraint-induced movement therapy, progressive resistance training) leads to significant improvements in upper-limb motor function, grip strength, and dexterity in pwMS [9,10]. Moreover, Bonzano et al. found that upper-limb motor rehabilitation positively impacts the brain’s white matter microstructures in pwMS [11].

During the last decade, various investigations have examined the use and efficacy of virtual reality (VR)-based intervention programs whose goal was to improve upper-limb functions in the neurological population (e.g., stroke survivors, Parkinson’s disease, cerebral palsy), including pwMS [12,13,14,15]. Rehabilitation via VR training tools allows repetitive, intensive, and task-specific practice in a controlled and safe environment. Moreover, it is believed that VR training is more advantageous (over standard care) in terms of patient engagement and motivation during practice, improving adherence and overall outcomes [16,17,18,19,20].

In accordance with the recent literature, practice with VR rehabilitation tools can improve motor function, dexterity, and hand grip strength in pwMS [12]. Cuesta-Gomez et al. found that VR-based training led to significant improvements in unilateral gross manual dexterity, fine manual dexterity, and coordination in pwMS with moderate–severe disability (EDSS scores of 3.5–7.5), together with high satisfaction and excellent compliance [21]. Recent studies have provided encouraging, although partly mixed, evidence as to the impact of immersive VR on the upper-limb function of pwMS. In fact, Bertoni et al. demonstrated that an immersive VR-based approach was useful in improving the gross manual dexterity in the less-affected limb of pwMS, although such improvement did not translate into the ability to carry out activities of daily living (ADL) or an improvement in fine hand dexterity, strength, or fatigue [22]. On the other hand, Pau et al. reported that this approach can significantly improve the speed and stability of the hand-to-mouth task, which is important for feeding and drinking [23].

Despite the rapid growth of VR-based approaches, there is still no clear consensus on which approach (immersive vs. non-immersive) is most effective in pwMS’s rehabilitation. Furthermore, no clear definition has been established for an optimum intervention duration and intensity needed to achieve a meaningful clinical difference. It is worth noting that in order to increase the efficacy of VR training in rehabilitation, Xie et al. recommended that the practice sessions should be based on real-life activities in a realistic environment [24]. Therefore, the goals of our study were: (1) to develop and validate a novel immersive VR system based on the practice of real-life activities in a kitchen environment in pwMS with upper-limb dysfunction and (2) to examine the safety, satisfaction and adherence of the new system in a small sample of pwMS with upper-limb dysfunction.

## 2. Materials and Methods

### 2.1. Development of the Immersive Virtual Kitchen Training System

The immersive virtual reality kitchen application designed by our research group in collaboration with the Italian company SJM Tech (https://www.sjmtech.net accessed on 20 April 2023) simulates daily-life activities, concentrating on the use of the upper limbs. Several demonstrations of the virtual kitchen can be found via the following link: https://doi.org/10.6084/m9.figshare.22652251 accessed on 20 April 2023. This application was developed using Unity3D (v.2019.4.29f1, Unity Technologies, San Francisco, CA, USA), a cross-platform game engine designed to build games for different platforms, making it the most widespread engine in the gaming sector. In particular, a series of custom scripts was created with the Auto Hand VR Physics interaction framework (Earnest Robot, http://www.earnestrobot.net accessed on 20 April 2023), a user-friendly VR interaction toolkit that automatically determines what position a hand should take when grasping an object. This toolkit was used to implement the virtual-space locomotion, object-grasping interactions, object interaction via pointers, 3D button interactions, and 3D body physics within the virtual space.

In our study, this application was created to run on a commercial VR headset (Meta Quest 2, formerly known as Oculus Quest 2) developed by Meta Platforms Inc. and released in 2020. The system, equipped with a singular, fast-switch LCD panel with a per-eye resolution of 1832 × 1920 and a refresh rate of up to 120 Hz, supports a physical interpupillary distance adjustment at 58 mm, 63 mm, and 68 mm. The Quest 2 also includes two controllers, thus allowing players to manage hand movements and actions within the simulation.

The virtual kitchen can be used both via the Oculus link (connected to a PC through a dedicated cable) or in a standalone mode (as an Oculus Quest application); thus, it can be downloaded as either an .exe or .apk file (depending on the destination platform) from a remote cloud system. Both solutions have advantages and disadvantages. Indeed, when used as an .apk application on Oculus Quest, the game experience is totally free from external disturbances (i.e., the need for cables) and the movements can be performed more easily. However, this gaming modality relies solely on the Oculus Quest hardware which, of course, is not as powerful as a computer; hence, its graphic limitations must be taken into account. In particular, in order to avoid image distortion or video glitches, the displayed scenes should not be too realistic and detailed. They are often portrayed as “cartoon-like”. By using simple geometries, it is easier to spot objects and help maintain the ease in performing tasks, even for individuals not very familiar with videogames.

**Figure 1:**
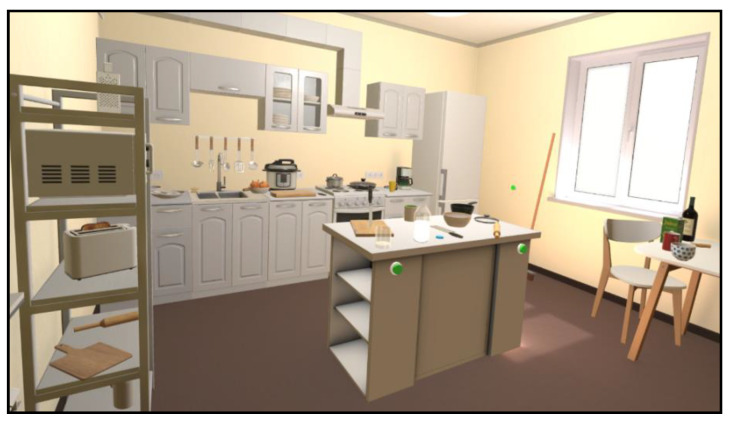
The virtual kitchen: global view.

Upon initiation, the application replicates a kitchen in which the user can see/move/use his/her hands. The room includes kitchen furniture, a sink, a countertop with a chopping board, household appliances (i.e., microwave oven, toaster, gas hob, electric oven, fridge, and coffeemaker), as well as tableware, cutlery, bowls, pans, bottles, and glasses (Figure 1). The kitchen is fully equipped with grocery items inside the fridge and on the table and countertop. The game mode enables the user to familiarize him/herself with the environment and also explore and manipulate every object present on the scene.

**Figure 2:**
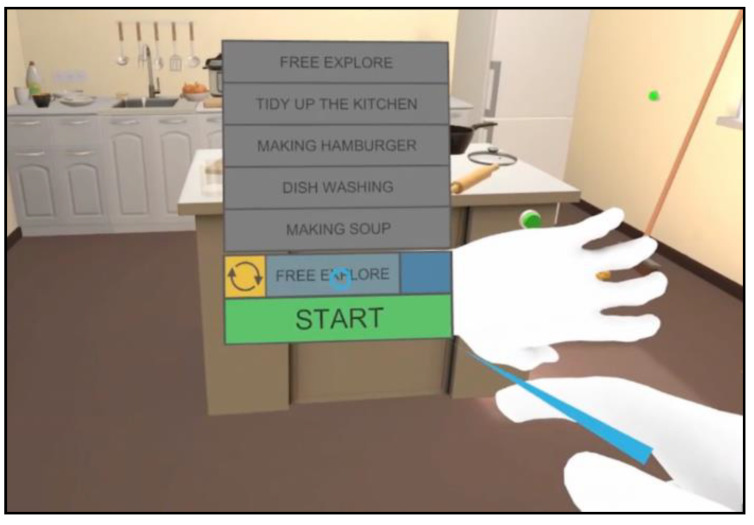
The menu activated by the virtual wristband.

When the user rotates their left wrist (i.e., looking at a wristwatch), a virtual wristband appears (Figure 2) from which it is possible to reset the current scene, select one of the five pre-determined tasks, begin the task, and check the elapsed time from the beginning of the task to the end.

**Figure 3:**
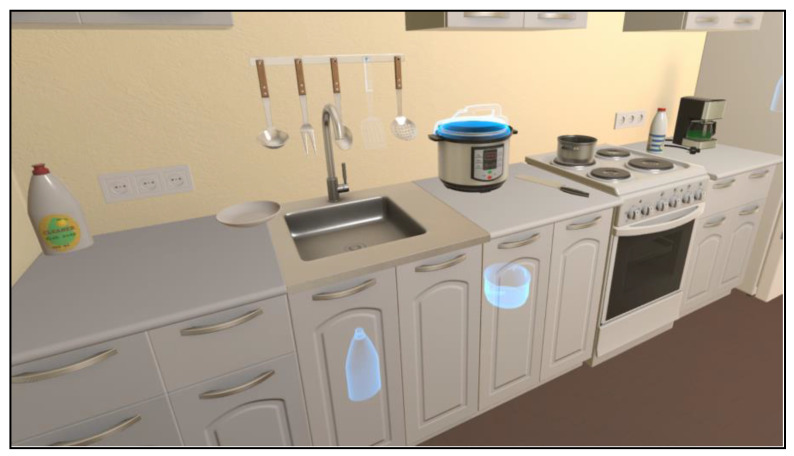
Tidying up the kitchen. The glowing silhouettes indicate the correct positions of the objects.

**Figure 4:**
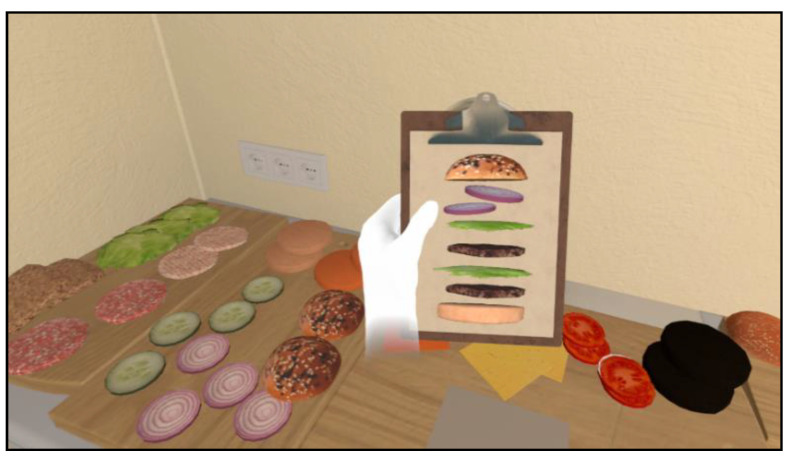
Preparing a hamburger before cooking.

**Figure 5:**
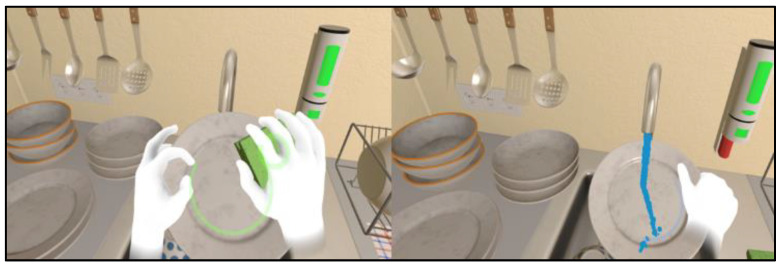
Dish washing.

**Figure 6:**
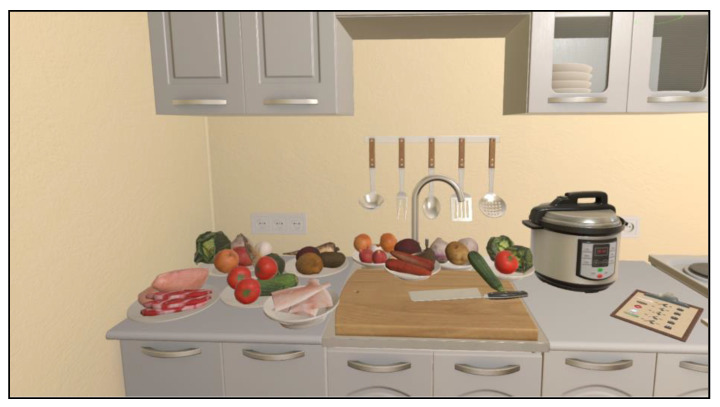
Preparation of soup before cooking.

There are five functional tasks of the application software:

- Free explore (default mode at the start of the application, previously described)
- Tidying up the kitchen (Figure 3). The aim of this routine was to tidy up the kitchen after a series of objects had been scattered throughout the scene and place them correctly into their rightful positions (highlighted by a shiny transparent outline). Each time the routine is started, the initial position of the objects required to reorder is randomly changed. The performance is evaluated according to the time needed to complete the task, displayed on the wristband. The task completion is accompanied by an acoustic signal.

- Preparing a hamburger before cooking (Figure 4). The user is required to place the different ingredients necessary to prepare a hamburger before cooking in the right order, as indicated by an electronic board located on the table. Each time the ingredient is properly placed, a checkmark appears on the board. The meat must be cooked before placing it in the sandwich. The correct amount of time needed to avoid overcooking is indicated by a yellow circle appearing on the meat during the cooking process. When the task has been correctly completed, a checkmark appears on the board accompanied by audio feedback.

- Dish washing (Figure 5). The user stands in front of the sink, which contains tableware needing to be cleaned. A sponge from the sink must be grasped and soaked with a liquid dish-washing detergent (taken from a dispenser near the sink) and water. Then, each dish, bowl, and mug must be soaped, rinsed, and placed in the correct section of the dish rack. The appropriate cleaning state is highlighted by a change of color in a light ring that appears on the dish. After several pieces of tableware have been washed, it might be necessary to add more detergent.

- Preparing soup before cooking (Figure 6). The goal of this task is to correctly prepare a soup following the sequence displayed on the board. In particular, the following steps must be completed: ingredient selection (meat, fish, and vegetables), chopping, positioning the ingredients into an electric slow-cooker, and cooking. Each time an ingredient has been properly chopped and placed in the pot, a checkmark appears on the board. A button is available to restore the ingredient supply when needed.

### 2.2. Study Design and Participants

This validation study was conducted at two established MS centers: (1) the Regional Center of Sardinia for Diagnosis and Treatment of Multiple Sclerosis (Binaghi Hospital, Cagliari, Italy) and (2) the Multiple Sclerosis Center, Sheba Medical Center, Tel-Hashomer, Israel. pwMS were recruited according to the following criteria: Inclusion: (1) diagnosis of MS according to the revised McDonald Criteria 2017 [25]; (2) aged 25–60 years old; (3) Expanded Disability Status Scale score  ≥  6 [26]; (4) ability to understand and execute simple instructions; (5) ability to place >0.5 pegs/s into the holes (=18 s) when performing the Nine-Hole Peg Test (NHPT). This score was selected due to its high discriminative and predictive ability in distinguishing ADL independence in pwMS [7]. Exclusion: (1) orthopedic and other neurological disorders affecting upper-limb movements (e.g., epileptic seizures); (2) contra-indication to physical activity (e.g., heart failure, severe osteoporosis); (3) moderate or severe cognitive impairments as indicated by a Mini-Mental State Examination [27] score  <  21; (4) pregnancy (self-reported); (5) severe uncorrected visual deficits; (6) MS clinical relapse or treatment with corticosteroid therapy within 90 days prior to enrollment; (7) started or stopped a disease-modifying therapy for MS within 90 days prior to enrollment; (8) received a course of physical or occupational therapy (home, outpatient, or inpatient) within the last 30 days; (9) other treatments that could influence the effects of the interventions. The study was conducted in accordance with the Declaration of Helsinki, and approved by the Institutional Review Board Committee (IRB) of ATS Sardegna, Italy and Sheba Medical Center, Tel-Hashomer, Israel (SMC-6408-19, 15 March 2020). All eligible participants provided written informed consent.

### 2.3. Study Protocol

This study, performed on a small sample of pwMS (n = 8) suffering from upper-limb dysfunction, was based on an 8-week intervention period (two sessions per week, length of session ~50 min) using the new immersive VR development system. The length and frequency of the intervention period is in accordance with other studies that have investigated upper-limb physical rehabilitation in pwMS [8]. Outcome measures related to the functionality of the upper limbs were collected on three occasions: T0 (baseline)—one week prior to the intervention; T1 (post-intervention)—within 1 week after completion of the intervention; and T2 (follow up)—a one month follow-up after completion of the intervention. During the follow-up period (T1–T2), the participants were instructed to continue their regular activities. The assessment of the safety, usability, and acceptance of the new system was collected at T1. Over the course of the 8-week intervention period, each kitchen scenario drill was performed in at least ten training sessions, ensuring a balanced and standardized selection for all participants. Modifications as to the practice complexity, intensity, and rest breaks between training tasks were tailored according to the patient’s ability and fatigability. The intervention program, including assessments, was performed at the two MS centers mentioned above by an occupational therapist experienced in neuro-rehabilitation.

### 2.4. Outcome Measures

#### 2.4.1. Measure of Safety, Usability, and Acceptance of the VR System

In order to evaluate the safety, usability, and acceptance of the new immersive virtual kitchen development system, we used the Suitability Evaluation Questionnaire (SEQ) designed specifically for VR systems [28]. The SEQ includes 14 questions, 13 graded on a 5-point Likert scale and a final yes/no question. Questions 1–7 are associated with enjoyment, sense of comfort and discomfort, feelings of success and control, realism, and easy-to-understand instructions. Questions 8–11 focus on dizziness/nausea symptoms, eye discomfort, disorientation/confusion symptoms, and sense of progress in rehabilitation. Questions 12 and 13 evaluate the difficulty of the task and usage of the system. The global score of SEQ ranges from 13 (poor suitability) to 65 (excellent suitability). SEQ has demonstrated a good internal consistency reliability [28].

#### 2.4.2. Assessment Tools Related to Functionality of the Upper Limbs

- Nine-Hole Peg Test (NHPT). The NHPT is recommended as a gold standard for measuring manual dexterity in pwMS [29] and has excellent psychometric properties as to its reliability; discriminant, concurrent, and ecological validity; detecting progression over time; and sensitivity to treatment. Briefly explained, the NHPT requires participants to repeatedly place nine pegs into nine holes, one at a time, as quickly as possible and subsequently, remove them from the holes. The total time needed to complete the task is then recorded. Two consecutive trials with the dominant hand are immediately followed by two consecutive trials with the non-dominant hand.
- Action Research Arm Test (ARAT). The ARAT is a 19-item observational measure used to assess upper extremity performance in terms of coordination, manual dexterity, and functioning in neurologic conditions, including pwMS [30]. The ARAT items are categorized into four subscales (grasp, grip, pinch, and gross movement) and are arranged in order of decreasing difficulty, with the most difficult task examined first. The task performance is rated on a 4-point scale, ranging from 0 (no movement) to 3 (movement performed normally).
- Manual Ability Measure-36 (MAM-36). The MAM-36 is a questionnaire referring to the ease or difficulty that a person experience unilateral and bilateral ADL tasks. During a semi-structured interview, the subject is asked to rate 36 unilateral and bilateral ADL tasks using a 4-point Likert scale. The MAM-36 has satisfactory psychometric properties and is recommended as an outcome measure for upper-limb function in pwMS [31].
- Health status questionnaire (SF-36). The Short Form-36 is one of the most widely used measures of health-related quality of life and has been shown to discriminate between subjects with different chronic conditions and between subjects with different severity levels of the same disease. The questionnaire addresses health concepts relevant to pwMS from the patient’s perspective. The availability of normative data makes the SF-36 useful for comparative purposes. There is substantial evidence of the validity of the SF-36 in pwMS [32].

## 3. Results

Ten pwMS with upper-limb motor dysfunction were recruited. Two participants did not complete the intervention program due to transport issues preventing arrival to the clinical center; therefore, the outcome measures were based on eight patients who completed the intervention phase including a follow-up evaluation. All participants were right-hand dominant with a secondary progressive type of disease and had completed at least 12/16 treatment sessions. Six (n = 75%) were female, the mean age was 56.4 (range 48–65), the median Expanded Disability Status Scale score was 6.5 (range 6.0–7.0), and the mean disease duration was 15.5 years (range 10–22). The demographic and clinical data of the eight participants who completed the intervention period are presented in Table 1.

Table 2 presents the scores of each participant who answered the SEQ questionnaire for VR systems. The mean scores for items 1–6 and 11 concerning the level of enjoyment, sense of comfort, feelings of success and control, realism, easy-to-understand instructions, and assisting in rehabilitation were between 4.0 and 4.6 (out of 5), indicating a high satisfaction. Regarding the general discomfort felt during practice (item 7), the mean score was 2.9 (S.D. = 1.55), indicating moderate comfort. The scores regarding eye discomfort, dizziness, nausea, and disorientation during practice (items 8–10) were between 2.8 and 1.3, indicating a low-to-moderate interference of the system. The participants reported that the system tasks and usage were relatively easy (items 12, 13).

Table 3 presents the scores of the outcome measures according to the study timeline. Although a trend was observed for minimal improvements in upper-limb functions, the significance level was not reached.

## 4. Discussion

Overall, we demonstrated that the novel immersive virtual kitchen training development system is feasible, safe, and well-accepted and can benefit pwMS suffering from upper-limb impairment. In accordance with our main findings, the participants rated their experience with the system as very good, with little discomfort during practice. Moreover, most patients felt that the virtual kitchen training system could assist in their rehabilitation. Yet, these findings were not supported by the upper-limb function and quality-of-life outcome measures. Non-significant differences between the pre- and post-evaluations were found in these measures. Nevertheless, this result is probably, at least partially, due to the small sample. It is also possible that the clinical tests routinely employed to assess upper-limb function are not fully suitable to capture the possible improvements associated with the use of this innovative approach. In this context, the use of quantitative techniques for human movement analysis (i.e., motion-capture systems and inertial measurement units), might provide useful data acquired during the execution of more ecological tasks.

A significant segment of our study concerned the development phase. Important decisions drawn from this period included the level of immersion, which functional upper-limb tasks needed to be practiced, and in what context. Our primary decision was to use fully immersive VR when images are presented through a head-mounted display. Fully immersive VR systems have several advantages compared to non-immersive devices such as a greater sense of presence, more interactivity, and a greater sense of immersion. The disadvantages are mainly physical discomfort for some users, e.g., the head-mounted display can be heavy and uncomfortable to wear for extended periods of time and the virtual environment can cause motion sickness or eye strain for others [33]. To our satisfaction, according to the participants’ responses, our novel fully immersive VR system created minimal physical discomfort; nevertheless, it is important to acknowledge that the number of pwMS who tested it was relatively low.

A further decision made during the development phase was to focus on ADL and upper-limb functional activities, generally limited in many pwMS with upper-limb impairments. We, therefore, focused on common activities requiring gross movements of the shoulder, elbow, and wrist joints in combination with manual dexterity. Interestingly, cooking ability has declined in pwMS [34]. Moreover, poor cooking abilities in pwMS have been found to be associated with cognitive decline [34]. For this reason, several kitchen practice drills (e.g., preparing a hamburger) were combined with different cognitive challenges (e.g., remembering the order of the ingredients). Cognition was not assessed after the intervention phase; hence, we cannot claim that our VR system improves cognitive abilities. To explore this interesting query, we are currently designing a larger RCT where cognition will be a primary outcome measure.

It is worth noting that we did not incorporate games into the virtual kitchen training software. The new development is based solely on functional tasks; consequently, it is not labeled as an exergaming device. Exergaming combined with VR for upper-limb rehabilitation has become popular during the last few years in various populations, including pwMS [22,35,36]. Although the use of exergaming for rehabilitation has several advantages (e.g., increased motivation and engagement) [37], several disadvantages warrant attention. First, for several patients the game might be too complicated, thus leading to a high percentage of failure, decreasing motivation to practice. Second, we dispute the assumption that all patients receiving rehabilitation prefer exercise via games compared to standard exercise. Some patients might even feel that the game scenario is childish. Moreover, many VR devices used in rehabilitation are based on commercial games, requiring specific movements for game success, and may not be related to the movements required during functional tasks, which is the main purpose of physical rehabilitation. For these reasons, we endeavored to develop a device that focuses solely on the practice of daily functional activities of the upper limbs, without any gaming distractions.

### Limitations of the Study and Future Directions

The present study adds significant information to the field of advanced technologies for upper-limb rehabilitation in pwMS. However, there are several weaknesses that deserve attention. First, the sample size of the pilot study was small; therefore, the statistical power to detect significant differences in the outcome measures between the three evaluation time points was limited. Nonetheless, a small size is usually sufficient for a safety and feasibility study, which was the main purpose of the current study. Importantly, a future RCT will examine the efficacy of the new system, comparing it with an active control group in a larger cohort of pwMS. A second limitation worth noting relates to the current immersive VR headset, including the two hand-held controllers. Incompatibility exists between the hand movements required to perform a task in the virtual kitchen with the actual hand movements on the hand-held controller. For example, in order to perform a grasp movement in the virtual kitchen environment, the user needs to press a specific button on the hand-held controller. This incongruity between the actual hand movements (i.e., pressing a button) and the different hand movements that appear in the virtual kitchen (i.e., grasping an object) requires a learning period and basic cognitive capabilities; therefore, it might be too complicated for patients with major cognitive deficits to perform. The good news is that this limitation will probably be solved in the near future since new VR systems use advanced controllers based on sensitive sensors. The new systems are able to capture slight movements from each digit and thumb [38]. Finally, we did not evaluate cognition as an outcome measure; consequently, we cannot claim that the virtual kitchen training system improves cognitive capabilities. We intend to include this measure in a future RCT.

## 5. Conclusions

The virtual kitchen training system is feasible and safe for upper-limb training in pwMS. Overall, the participants rated their experience as very good, with a few feeling discomfort during the practice. Furthermore, most patients believed that the virtual kitchen training system could assist in their rehabilitation. These findings will pave the way for future RCTs to examine the benefits of the system by comparing it with standard care, thus improving the functionality of the upper limbs in pwMS and/or other clinical populations. Furthermore, it would be noteworthy to compare training at a clinical facility compared to training via telehealth, both monitored by a physical therapist. Additionally, an assessment of the system by healthcare professionals in the field of neurorehabilitation should be undertaken. Finally, future research should examine the psychometric values of the outcome measures produced by the system in patients with upper-limb dysfunctions.

## Figures and Tables

**Table 1:** Demographics (mean value, S.D.) and clinical measures of the study sample (n = 8).

Characteristic	Value (S.D.)	Range
Age (years)	56.4 (5.7)	48–65
Female, n (%)	6 (75%)	---
Type of MS		
Relapsing–remitting, n	0	---
Secondary progressive, n	8	---
Median EDSS (score)	6.5	6.0–7.0
Disease duration (years)	15.5 (7.2)	10.0–22.0
Right-hand dominant, n	8	---
Height (cm)	161.3 (8.8)	152.0–181.0
Weight (kg)	53.4 (4.8)	46.0-61.0

**Table 2:** Patient scores (mean value, S.D.) of the Suitability Evaluation Questionnaire for VR systems.

Question	Response (1 = Not at All, 5 = Very Much)
1. How much did you enjoy your experience with the system?	4.4 (0.52)
2. How much did you sense that you were present in the environment?	4.6 (0.52)
3. How successful were you in the system?	4.3 (1.04)
4. To what extent were you able to control the system?	4.0 (0.93)
5. How real is the virtual environment of the system?	4.1 (0.83)
6. Is the information provided by the system clear?	4.6 (0.52)
7. Did you feel discomfort during your experience with the system?	2.9 (1.55)
8. Did you experience dizziness or nausea during your practice with the system?	2.8 (1.49)
9. Did you experience eye discomfort during your practice with the system?	1.3 (0.46)
10. Did you feel confused or disoriented during your experience with the system?	1.4 (0.52)
11. Do you think that this system will be helpful for your rehabilitation?	4.3 (0.46)
12. Did you find the task/s difficult?	2.0 (0.93)
13. Did you find the devices of the system difficult to use?	1.8 (1.04)
14. Did you feel uncomfortable during the task/s? (Yes/No)	No (7), Yes (1)

**Table 3:** Outcome measures according to time (mean, S.D.).

Outcome Measure	Baseline	Post-Intervention	One-Month Follow-Up	*p*-Value
NHPT				
Dominant	28.6 (5.3)	26.7 (4.2)	28.2 (4.1)	0.139
Non-dominant	37.8 (12.2)	37.3 (12.6)	40.1 (11.8)	0.067
ARAT				
Dominant	56.4 (0.7)	53.6 (1.6)	56.0 (1.2)	0.392
Non-dominant	53.5 (5.3)	55.9 (2.4)	54.9 (5.4)	0.353
MAM-36	110.1 (18.0)	116.1 (22.4)	112.8 (25.4)	0.135
SF-36	38.9 (10.3)	46.6 (6.5)	46.4 (12.9)	0.505

Nine-Hole Peg Test (NHPT); Action Research Arm Test (ARAT); Manual Ability Measure-36 (MAM-36); Health status questionnaire (SF-36).

## Data Availability

The dataset used during the current study will be available from the corresponding author upon reasonable request.
